# Cellulose Nanofibers from a Dutch Elm Disease-Resistant *Ulmus minor* Clone

**DOI:** 10.3390/polym12112450

**Published:** 2020-10-23

**Authors:** Laura Jiménez-López, María E. Eugenio, David Ibarra, Margarita Darder, Juan A. Martín, Raquel Martín-Sampedro

**Affiliations:** 1 Forestry products Department, Forest Research Centre, INIA, Ctra de la Coruña Km 7.5, 28040 Madrid, Spain; laura.jimenez.lopez@csic.es (L.J.-L.); mariaeugenia@inia.es (M.E.E.); ibarra.david@inia.es (D.I.); 2 Instituto de Ciencia de Materiales de Madrid (ICMM), Consejo Superior de Investigaciones Científicas (CSIC), Sor Juana Inés de la Cruz 3, Cantoblanco, 28049 Madrid, Spain; darder@icmm.csic.es; 3 Departamento de Sistemas y Recursos Naturales, ETSI Montes, Forestal y del Medio Natural, Universidad Politécnica de Madrid, c. José Antonio Novais 10, 28040 Madrid, Spain; juan.martin.garcia@upm.es

**Keywords:** *Ulmus minor*, cellulose nanofibers, TEMPO-mediated oxidation pretreatment, mechanical pretreatment, residual lignin

## Abstract

The potential use of elm wood in lignocellulosic industries has been hindered by the Dutch elm disease (DED) pandemics, which have ravaged European and North American elm groves in the last century. However, the selection of DED-resistant cultivars paves the way for their use as feedstock in lignocellulosic biorefineries. Here, the production of cellulose nanofibers from the resistant *Ulmus minor* clone Ademuz was evaluated for the first time. Both mechanical (PFI refining) and chemical (TEMPO (2,2,6,6-tetramethylpiperidine-1-oxyl radical)-mediated oxidation) pretreatments were assessed prior to microfluidization, observing not only easier fibrillation but also better optical and barrier properties for elm nanopapers compared to eucalyptus ones (used as reference). Furthermore, mechanically pretreated samples showed higher strength for elm nanopapers. Although lower nanofibrillation yields were obtained by mechanical pretreatment, nanofibers showed higher thermal, mechanical and barrier properties, compared to TEMPO-oxidized nanofibers. Furthermore, lignin-containing elm nanofibers presented the most promising characteristics, with slightly lower transparencies.

## 1. Introduction

The field elm (*Ulmus minor* Mill.) is a hardwood tree native to southern Europe. It thrives well in riparian habitats and floodplains of the Mediterranean basin, and has an outstanding tolerance to wind, mechanical damage, poor soils, frost, flooding and moderate summer drought [1]. Historically, this species has been used for different purposes, such as living supports of grapevines, medicine, food, cattle fodder, fibers, construction and firewood [1,2]. Moreover, due to its beauty and stress tolerance it has been widely used as a shade tree in urban areas. However, its high susceptibility to the alien fungi *Ophiostoma ulmi* and *O. novo-ulmi*, causal agents of the Dutch elm disease (DED), has provoked the disappearance of most adult *U. minor* trees in the last century [1]. The enormous impact of this disease, which also affected other European and North American elm species, has hindered the use of elm in forestry and associated industry. For these reasons, the search of DED-resistant cultivars has been of major interest for different research institutions in Europe and North America [1,2]. After the selection of several *U. minor* genotypes resistant to DED in Spain [3], the first pilot elm reforestation projects were recently initiated [1]. The wide ecological range of this species and its remarkable growth rate and resprouting capacity make it an interesting choice as feedstock for wood products. The search of new biomass applications for these DED-resistant cultivars could also contribute to boost elm reintroduction in forestry.

Among the DED-resistant cultivars registered in Spain for forest use, the clone Ademuz have shown an outstanding adaptation to different environments and salient growth characteristics [4]. The potential use of this clone as raw material in lignocellulosic biorefineries has been previously studied by our group [5]. In this previous work, the production of fermentable sugars and the revalorization of lignin residues as biochar for energy and environmental applications were studied. However, the production of nanocellulose has not been previously evaluated neither from this resistant clone nor other elm species, as far as we know. Nanocellulose, including both cellulose nanofibers and cellulose nanocrystals, is one of the most promising bioproducts that can be obtained in a biorefinery, due to its versatility and broad range of applications including nanocomposites, microelectronics, gas-barrier films, food-packaging, cosmetics, drug delivery systems, insulating and flame-resistant materials and other high-tech and high-performance materials [6,7,8]. Some of the appealing properties of nanocellulose are its low density, high strength, high optical transparency, large surface area, non-toxicity, and versatile surface chemistry [8,9]. Cellulose nanofibers are obtained by a high-shear mechanical treatment which fibrillates cellulose fibers into high-aspect ratio nanofibers containing both amorphous and crystalline cellulose domains. This mechanical treatment is usually performed in a high-pressure homogenizer or a microfluidizer, after pulp refining [10,11]. It can be also combined with chemical (TEMPO (2,2,6,6-tetramethylpiperidine-1-oxyl radical)-mediated oxidation, carboxymethylation, etc.) or enzymatic (mainly with endoglucanases) pretreatments in order to facilitate fibrillation and reduce energy consumption [8,9,10]. Both the selection of the production process and the raw material highly influence the final properties of the obtained cellulose nanofibers [9,10]. 

The main objective of this work was to evaluate the suitability of clone Ademuz as raw material for the production of cellulose nanofibers. Two different pretreatments—mechanical (PFI refining) and chemical (TEMPO-mediated oxidation)—were applied before microfluidization of bleached and unbleached elm pulps. Similar processes were applied to commercial bleached eucalyptus pulp for comparison in order to evaluate the effect of the selected raw material on the final nanofiber properties. Furthermore, although both pretreatments have been previously studied for several raw materials [8,9,10], only few articles include the comparison of both pretreatments using the same pulp as starting material [7,12,13]. In this way, the present work allows a deeper evaluation of the pretreatment effect on the nanofiber properties. Finally, by comparing the production and characteristics of cellulose nanofibers from both unbleached and bleached elm pulps, the effect of residual lignin content can be also elucidated. 

## 2. Materials and Methods 

### 2.1. Raw Materials and Chemicals

The plant material consisted of two replicates of the *U. minor* clone Ademuz, registered in Spain as resistant to DED [3]. These replicates were obtained from aerial cuttings in 2014 and grown in a field experimental plot at Valsaín National Center for Forest Genetic Resources, located in Segovia (Spain). In October 2018, the two trees were cut at the base of the main stem and kindly provided by the Valsaín Center to carry out this work. At the time of harvesting, the trees were around 4 m in height and 3.5 cm in diameter at breast height. Tree stems were debarked and cut into small chips of 0.5–1.5 cm length and 1–2 mm wide. Chemical composition of this raw material was determined following the procedure described in Section 2.6.1. Finding: 3.0% ethanol extractive, 19.3% Klason lignin, 1.6% acid soluble lignin, 48.6% glucan, 16.2% xylan and 4.9% acetyl groups.

*Eucalyptus globulus* bleached pulp was kindly provided by La Montañanesa pulp mill (Lecta Group, Zaragoza Spain). This commercial pulp was obtained using a standard kraft pulping followed by oxygen delignification and an Elemental Chlorine Free (ECF) bleaching sequence (DEPD: Chlorine dioxide stage, alkali extraction with hydrogen peroxide, and final chlorine dioxide stage). This never-dried pulp was stored at −18 °C until use.

All chemicals used in this work were reagent-grade and obtained from Merck (Barcelona, Spain) or Sigma–Aldrich (Madrid, Spain).

### 2.2. Production of Unbleached and Bleached Elm Pulps

Elm chips were subjected to kraft pulping in a 15-L batch reactor with a jacket-type electrical heater controlled by a computer and a rotating axle to ensure proper agitation. Cooking conditions were: 16% active alkali, 21% sulfidity, 90 min to maximum temperature, 90 min at maximum temperature (165 °C) and a liquor-to-wood ratio of 6 L/kg. Resulting pulp was called: unbleached elm pulp (UnBl-Elm).

Before bleaching, UnBl-Elm pulp was subjected to oxygen delignification in the same reactor. Conditions of this treatment were 98 °C, 60 min, 0.6 MPa of oxygen pressure, 1.5% NaOH over dry pulp (o.d.p.), 0.5% MgSO_4_ o.d.p. and 10% (*w/v*) consistency. Then, a chelation stage was performed to remove heavy metals in pulp, which could reduce the efficiency of subsequent hydrogen peroxide bleaching. This stage was carried out at 85 °C for 60 min with pH 5–6, 0.3% diethylenetriaminepentaacetic acid (DTPA) and 10% (*w/v*) consistency. Finally, a two-stage hydrogen peroxide bleaching was conducted. Conditions of the first bleaching stage were: 3% H_2_O_2_ o.d.p., 2% NaOH o.d.p., 0.1% Mg_2_SO_4_ o.d.p., 10% (*w/v*) consistency and 105 °C over 140 min. The second bleaching stage was carried out under the same conditions but at 98 °C over 180 min. Pulp was washed and filtered after each treatment in the bleaching sequence. Final bleached elm pulp (Bl-Elm) was stored at 4 °C until further use.

### 2.3. Production of Cellulose Nanofibers (CNFs) by Mechanical Methods

Both unbleached and bleached elm pulps were used to produce cellulose nanofibers by mechanical methods. Resulting CNF samples were called CNF-UnBl-Elm and CNF-Bl-Elm from unbleached and bleached pulps, respectively. Furthermore, CNF samples by mechanical methods were also prepared from the commercial never-dried bleached eucalyptus pulp, as reference raw material: CNF-Bl-Eu.

#### 2.3.1. Mechanical Pretreatment: Refining in PFI Mill

Mechanical pretreatment consisted of a refining treatment in PFI mill until a Schopper–Riegler degree (SR) above 90° was reached. Previously, pulps were soaked in diluted HCl solution (pH = 2, 10% (*w/v*) consistency, 20 min, mechanical agitation) and intensively washed with water to remove heavy metals, if present. Then, fibers were exchanged to their sodium form as described by Solala et al. [14] to enhance fibrillation efficiency. Briefly, 10% (*w/v*) fiber suspensions were prepared in 5-mM NaHCO_3_ solution (20 min with mechanical agitation) and washed with deionized water until filtrate conductivity was lower than 20 µS/cm. Finally, 30 g of 10% (*w/v*) exchanged pulp was treated in the PFI mill applying between 13,000 and 17,550 revolutions, depending on the pulp sample, until reaching 90° SR. 

In order to further improve fibrillation and to avoid clogging in the microfluidizer, 2% (*w/v*) refined pulp suspension was subjected to extensive mixing in an Ultra-Turrax disperser (T25, IKA): 10 min at 18,000 rpm followed by 10 min at 22,000 rpm.

#### 2.3.2. Microfluidization

The never-dried refined pulps were processed in a high-pressure fluidizer (Microfluidizer M-110EH, Microfluidics Corp., Westwood, MA, USA). Fiber suspensions (2% *w/v*) were passed thirteen times through an intensifier pump that increased the pressure, followed by one or two interaction chambers, which defibrillated the fibers by shear forces and impacts against the channel walls and colliding streams. The first six passes were carried out using only one interaction chamber of 200 µm (3 passes at 1200 bar and 3 more at 2000 bar), while the rest of the steps were performed adding a second chamber of 100 µm (3 passes at 1200 bar and 4 passes at 2000 bar). The obtained slurry of cellulose nanofibers was stored at 4 °C until use.

### 2.4. Production of TEMPO-Mediated Oxidized Cellulose Nanofibers (TOCNFs)

Never-dried bleached elm pulp was used to produce cellulose nanofibers with chemical pretreatment (TEMPO-mediated oxidation) prior to microfluidization. Resulting cellulose nanofibers were called TOCNF-Elm. In addition, TEMPO-mediated oxidized cellulose nanofibers were also prepared from the commercial never-dried bleached eucalyptus pulp, as reference: TOCNF-Eu.

#### 2.4.1. Chemical Pretreatment: TEMPO-Mediated Oxidation

Bleached pulp (45 g) was first acid-washed to remove heavy metals, as described in Section 2.3.1. Then, fibers were suspended in 4500 mL of deionized water containing 0.016 g/g o.d.p. of TEMPO (2,2,6,6-tetramethylpiperidine-1-oxyl radical) and 0.1 g/g o.d.p. of NaBr. TEMPO-mediated oxidation was started by adding 10 mmol/g o.d.p of NaClO. The reaction was carried out at room temperature. During oxidation, the pH of the slurry was maintained at 10 by adding 0.5-M NaOH until no decrease in pH was observed. TEMPO-oxidized fibers were filtered and washed with deionized water until neutral pH. 

#### 2.4.2. Microfluidization

After chemical pretreatment, the 2% TEMPO-oxidized fiber suspensions were treated in the high-pressure fluidizer mentioned above. Oxidized fibers were passed one time through one chamber of 200 µm (1000 bar), followed by three steps passing through two sequential chambers of 200 and 100 µm (one pass at 1000 bar and two passes at 2000 bar). The slurry of cellulose nanofibers obtained was stored at 4 °C until use.

### 2.5. Nanopaper Preparation

Cellulose nanofiber films (nanopapers) were prepared from both mechanical (CNFs) and TEMPO-oxidized nanofibers (TOCNFs). Suspensions of 0.2% (*w/v*) cellulose nanofibers were prepared and stirred at room temperature overnight. Then, 14.0 or 38.9 mL of suspensions were poured into 45- or 90-mm polystyrene Petri dishes, respectively, and dried at 30 °C and 50% relative humidity (RH) for several days. 

### 2.6. Characterization of Pulps, Cellulose Nanofibers and Nanopapers

#### 2.6.1. Chemical Composition

Chemical composition of raw material, unbleached and bleached pulps was determined according to NREL/TP-510-42618: Laboratory Analytical Procedures for determination of structural carbohydrates and lignin in biomass [15]. In brief, extractives were estimated as the soluble fraction after extensive Soxhlet extraction with ethanol. Then, carbohydrate composition was determined by a quantitative acid hydrolysis in two steps performed over the free-extractive samples. An Agilent Technologies 1260 HPLC with refractive index detector (Agilent, Waldbronn, Germany) was employed to quantify carbohydrate concentration in the hydrolyzed liquids, using an Agilent Hi-PlexH column at 65 °C, with 5-mM sulfuric acid as mobile phase (0.6 mL min^−1^). Finally, Klason lignin was calculated as the solid residue left after the acid hydrolysis and acid soluble lignin was determined by UV–Visible spectrometry at 205 nm using a Jasco V-500 spectrophotometer (Jasco, Japan). 

#### 2.6.2. Carboxylate Content

Carboxylate contents of the different cellulose nanofiber samples were determined by the conductivity titration method [16] with some modifications. In brief, 0.1 g of dry pretreated pulp (TEMPO-oxidized or refined pulps) was dispersed in 500 mL of 1-mM NaCl solution, adjusting the pH to 2.5–3 by adding 0.1-M HCl. This suspension was stirred at 25 °C under nitrogen atmosphere over 30 min to exchange the sodium cations bound to the carboxyl groups by hydrogen ions. Then, it was titrated by adding 0.05-M NaOH. Conductivity was recorded during titration using a Pt conductivity cell 50–70 and GLP 31 conductivity meter from Crison (L’Hospitalet de Llobregat, Spain), and three regions were observed: the first one due to the presence of a strong acid (excess of HCl), the second one corresponds to the volume of NaOH required to neutralize the weak acids (carboxylic groups), and the third one due to NaOH excess. Carboxylate content (µmol/L) was determined according to the following equation:

(1) $$Carboxylate\;content=\frac{c\cdot \left(V_{2}\;-\;V_{1}\right)}{w}$$

where *V*_1_ and *V*_2_ are the NaOH volume (L) consumed at the first and second intersection point, respectively; *c* is the NaOH concentration (µmol/L) and *w* is the sample dry weight (g).

#### 2.6.3. Fibrillation Yield

Cellulose suspensions after microfluidization were diluted to 0.1% (*w/v*) and centrifuged at 4500 rpm for 20 min in order to separate nanofibrillated material (in supernatant) from non-fibrillated and partially fibrillated fibers (in sediment) [17]. Sediment was then dried to a constant weight at 104 °C. Finally, fibrillation yields were calculated as the relation between the weight of nanofibers in the supernatant (calculated by subtraction) and the weight of centrifuged cellulose. 

#### 2.6.4. Zeta-Potential Measurement

The colloidal stability of the cellulose nanofiber suspensions was determined by zeta-potential measurements. Nanofiber suspensions of 0.025% (*w/v*) were prepared in deionized water and a NanoBrook 90Plus PALS from Brookhaven Instruments (Holtsville, NY, USA) was used to this end. 

#### 2.6.5. Fourier Transform Infrared (FTIR) Spectroscopy

FTIR spectra of the different cellulose nanofibers were obtained using a JASCO FT/IR 460 Plus spectrometer, equipped with an accessory single reflection diamond. Spectra were obtained in a spectral range of 4000–600 cm^−1^, operating with a resolution of 1 cm^−1^ and 400 scans. 

#### 2.6.6. X-Ray Powder Diffraction (XRD) Analysis

Cellulose nanofibers were analyzed by X-ray powder diffraction using a Bruker D8 Advance diffractometer (Bruker, Billerica, MA, USA) equipped with a Cu anode (CuKα radiation) and Ni filter. Measurements were performed with a goniometer speed of 1 s per step, and a step size of 0.04 in the range of 2θ diffraction angle from 3° to 45°. Crystallinity index (CrI) of each sample was calculated based on the Segal method (Equation (2)):

(2) $$CrI=\frac{I_{200}-I_{AM}}{I_{200}}\cdot 100$$

where *I*_200_ is the height of the crystalline (200) peak and *I_AM_* is the height of the amorphous intensity.

The average size of the nanocrystallites (CrS) was also calculated according to the equation of Scherrer (Equation (3)) using the (200) reflection:

(3) $$CrS=\frac{k\cdot \lambda }{\beta \cdot cos\theta }$$

where *k* is the correction factor (0.9), *λ* is the radiation wavelength (0.15418 nm), *β* is the width of the peak at the half of maximum height (in radians), and θ is the diffraction angle of the peak.

#### 2.6.7. Thermogravimetric Analysis (TGA)

Thermal stability of the different cellulose nanofibers was determined by TGA measurements using a SDT-Q600 Thermogravimetric analyzer (TA Instruments, New Castle, DE, USA). Experiments were carried out under N_2_ atmosphere (flux of 100 mL min^−1^) from room temperature to 700 °C at a heating rate of 10 °C min^−1^.

#### 2.6.8. Mechanical Properties 

Mechanical properties of nanopapers were evaluated by a tensile test using a Model 3345 Instron Universal Testing Machine (Instron Engineering Corporation, Norwood, MA, USA). Nanopapers were cut into rectangular test pieces (80 × 10 mm). Initial grip separation and crosshead speed were fixed to 40 and 4 mm min^−1^, respectively. At least 4 measurements were carried out for each nanopaper. Young’s modulus (GPa), tensile strength (MPa) and elongation at break (%) were determined from stress–strain curves. Taking into account the apparent density of each nanopaper, the specific Young modulus (MN m Kg^−1^) and tensile index (kN m Kg^−1^) were also calculated. 

#### 2.6.9. Water Vapor Sorption Isotherms

Water vapor sorption isotherms were measured using dynamic water vapor sorption equipment, Aquadyne DVS (Quantachrome Instruments, Boynton Beach, FL, USA). Around 10 mg of nanopaper was purged at 80 °C until the sample weight remained constant. Mass changes due to water adsorption or desorption were recorded at 25 °C in the range of relative humidity from 0% to 95%. 

#### 2.6.10. Water Vapor Permeability

The water vapor permeability (WVP) and the water vapor transmission rate (WVTR) of the different nanopapers were determined gravimetrically based on ASTM E98-95 standard (procedure for desiccant method). The nanopapers were mounted in the test cells using aluminum foil masks, with an inner diameter of 1 cm. Test cells were filled with dry silica-gel and sealed before being placed in a humidity chamber at 75% relative humidity and room temperature. Test cells were weighed each 24 h over at least 4 days. Experiments were carried out by triplicate for each nanopaper. 

The slopes of the weight gain vs. time curves were used to calculate WVTR, by dividing the slope by the exposed film area. Permeability (WVP) was determined taking into account the thicknesses of the nanopapers (L, expressed in mm) and the partial water vapor pressure difference across both sides of the film (ΔP, in kPa), according to the following equation:

(4) $$WVP=\;\frac{\left(WVTR\cdot L\right)}{∆P}$$

#### 2.6.11. Optical Transmittance and Transmission Haze

Optical transmittance of cellulose nanofiber suspensions and nanopapers was studied using a UV-Spectrophotometer (Shimadzu, UV1201 model, Kyoto, Japan). For nanofiber suspensions, the slurry obtained after microfluidization was diluted to 0.1% (*w/v*) and placed into a quartz cuvette (1 cm path length), measuring the transmittance from 260 to 800 nm. A quartz cuvette filled with deionized water was used as the reference. 

For studying optical properties of nanopapers, the UV-Spectrophotometer was equipped with an integrating sphere. Both total transmittance and transmission haze of the transparent nanopapers were determined between 200 and 900 nm. 

## 3. Results and Discussion

### 3.1. Chemical Composition of Initial Pulps

Unbleached elm pulp (UnBl-Elm) was obtained after kraft pulping of the DED-resistant clone Ademuz. Further oxygen delignification and bleaching were carried out to obtain bleached elm pulp (Bl-Elm), reducing the lignin content from 2.5% to 1.4% (Table 1). A different behavior of unbleached and bleached elm pulps during production of nanofibrillated cellulose would provide an estimation of the lignin content effect on fibrillation and the final nanofiber properties, as other authors have reported using other raw materials [14,18,19]. Nevertheless, it should be taken into account that other non-cellulose components were also reduced during oxygen delignification and bleaching, such as ethanol extractives (from 1.3% to 0.9%) and hemicelluloses (mainly xylan, from 18.4% to 17.0%) (Table 1). 

Compared to a commercial eucalyptus pulp (Bl-Eu), used herein as reference material for cellulose nanofiber production, Bl-Elm pulp presented a similar lignin content (1.3% vs. 1.4%), but higher hemicellulose content (19.3% vs. 17.0%) (Table 1). This variance is mainly due to differences in the pulp production (pulping and bleaching) processes, since both woody raw materials presented similar hemicellulose content: xylan content of 15.6–16.6% for *Eucalyptus globulus* [20,21] and 16.2% for clone Ademuz. Nevertheless, since the same pretreatments and fibrillation steps were applied to Bl-Elm and Bl-Eu pulps, the comparison of both samples during nanofiber production and the final nanofibers’ properties would reveal the effect of the selected raw materials.

### 3.2. Production of Cellulose Nanofibers: Properties of Nanofiber Suspensions

Before fibrillation, a mechanical or chemical pretreatment was carried out to reduce the clogging and the energy consumption in the microfluidizer [8,10]. Both unbleached and bleached pulps were subjected to mechanical pretreatment consisting of PFI refining. However, only bleached pulps were subjected to chemical pretreatment. If TEMPO-mediated oxidation pretreatment were performed over unbleached pulp, part of the NaClO added would be consumed in oxidation and the degradation of lignin, reducing the efficiency of the pretreatment [22]. 

The mechanical pretreatment by PFI refining causes significant physical changes. The main ones are: internal fibrillation (breakage of inner bonds and swelling), external fibrillation (pilling off the fibrils from the fiber surface causing an increase in the specific surface area) and production of fines and shortening of the fibers [23]. Overall, fibers become more flexible and soft, while at the same time the specific surface area and volume are significantly increased, favoring fibrillation in the subsequent mechanical treatment (microfluidization) aimed at obtaining cellulose nanofibers [10]. On the contrary, no significant changes in chemical composition and functional groups are expected during refining, although slight variations can occur in the distribution of surface chemical compositions [23]. Thus, differences in carboxylate groups content among mechanically pretreated CNF samples (Table 2) can be assigned to differences among the initial pulps. A direct relation between carboxylate and hemicellulose contents was found—carboxylate groups being present in non-oxidized samples is mainly due to the presence of uronic acid in 4-O-methylglucuronoxylan, which is the main hemicellulose in hardwoods [24]. 

On the other hand, TEMPO-oxidation pretreatment causes the selective conversion of C6 primary hydroxyl groups of cellulose to carboxylate groups via the C6 aldehyde groups [6]. Thereby, TOCNF samples presented much higher carboxylate contents (1043–1178 µmol/g) (Table 2). The introduction of these negative groups in the surface of the fibrils causes repulsion between adjacent cellulose fibrils due to electrostatic forces. Consequently, it facilitates the disruption of fibers into nano- and microfibrils in the subsequent microfluidization step [6,8]. 

Due to the different effects caused by each pretreatment, important differences were found in nanofibrillation yields: 45–61% for CNF samples and 100% for TOCNF samples. These values indicated a high presence of microscopic fibers, instead of nanofibers, and their fragments in the suspension were obtained after microfluidization of mechanically pretreated samples, which caused higher light scattering resulting in less transparent CNF suspensions compared to TOCNF (transmittance of 14–19% and 77–93%, respectively, at 600 nm). The high carboxylate groups content of TOCNF also contributed to obtain stable nanofiber suspensions, due to electrostatic repulsion, as indicated the high zeta-potential of both TOCNF-Elm and TOCNF-Eu samples (−68 mV). Similar values of zeta-potential (~−75 mV) have been reported for TOCNF with different carboxylate groups content (from 520 to 1690 µmol/g), indicating a similar density of carboxylic groups on the fibril surface of the different TOCNF samples, probably because almost all the C6 primary hydroxyls exposed on the entire surface of cellulose microfibrils were converted to sodium carboxylate groups [6,25]. However, the low number of negative groups in the fibril surface of mechanically pretreated samples did not avoid fibril aggregation and flocculation (zeta-potential values close to 0 mV). Only CNF-Bl-Elm suspension showed a zeta-potential close to −30 mV (indicating colloidal stability). This sample presented the highest nanofibrillation yield (61%) and the highest transmittance at 600 nm (19%), among mechanically pretreated samples. Therefore, its higher colloidal stability could be related to a lower amount of non-fibrillated and partially fibrillated fibrils, which could easily flocculate, among other possible reasons.

On the other hand, the lower nanofibrillation yield obtained for CNF-UnBl-Elm (50%) compared to CNF-Bl-Elm (61%), could be due to the higher lignin content in unbleached fibers, promoting a more crosslinked and relatively hydrophobic structure which can hinder swelling and fibrillation [19]. Accordingly, several authors have reported that the removal of lignin and hemicellulose is essential to efficiently isolate cellulose nanomaterials [9,10]. However, other authors have reported an enhancement of fibrillation for lignin-containing fibers [11,14,18,19]. These authors attributed the ease of fibrillation to lignin acting as an antioxidant. The radical scavenging ability of lignin stabilizes cellulosic mechano-radicals formed during microfluidization, preventing broken covalent bonds from being formed again and resulting in less pronounced crosslinking of cellulose, which allows better deconstruction of the fibrils [11,14,19]. Indeed, when only the nanofibrillated fraction of CNF samples was studied by Atomic Force Microscopy (AFM) (removing non-fibrillated and partially fibrillated fibrils by centrifugation), thinner and shorter nanofibers were observed for CNF-UnBl-Elm (diameter of 3.7 ± 0.7 nm and length of 370 ± 14 nm) compared to CNF-Bl-Elm (5.9 ± 1.7 nm and 1237 ± 680 nm), as is indicated in Supplementary Materials (Figure S1). These results could indicate that, in spite of the presence of a higher amount of non-fibrillated or partially fibrillated fibrils in CNF-UnBl-Elm (lower nanofibrillation yield), once nanofibrils are individualized, lignin-containing fibrils suffer more intensive fibrillation achieving better deconstruction of the fibrils likely due to the antioxidant action of lignin.

The effect of raw material in fibrillation can be evaluated by comparing TOCNF-Elm with TOCNF-Eu and CNF-Bl-Elm with CNF-Bl-Eu. In both cases, lower fibrillation was achieved for eucalyptus (lower nanofibrillation yield and/or lower transmittance, Table 2). Furthermore, AFM results from the study of nanofibrillated fractions (Supplementary Materials, Figure S1) indicated thinner nanofibers in elms samples: lower diameter and narrower diameter distributions (2.6 ± 0.7 nm vs. 5.0 ± 1.4 nm for TOCNF-Elm and TOCNF-Eu, respectively, and 5.9 ± 1.7 nm vs. 6.1 ± 2.7 nm for CNF-Bl-Elm and CNF-Bl-Eu, respectively). The higher carboxylate content of TOCNF-Elm compared to TOCNF-Eu surely contributed to the higher fibrillation [6]. However, it does not justify the differences found in mechanically pretreated samples (CNF). Chemical composition of Bl-Elm and Bl-Eu pulps also does not explain these results, since higher hemicellulose content would contribute to enhance fibrillation by inhibiting fibril coalescence and hornification [9,19], but elm pulp showed slightly lower hemicellulose content than eucalyptus pulp (Table 1). According to several authors, the geometries and dimensions of cellulose nanofibers strongly depend on the sources and likely reflect their ultrastructure in the specific raw material [8,9]. This way, *U. minor* has libriform fibers (maximum diameter 10–15 μm), with irregular shapes and wavy arrangements, while *E. globulus* also has libriform fibers (maximum diameter 10–12 μm) but they are polygonal, with slightly wavy shapes and intertwined arrangements [26].

### 3.3. FTIR Analysis

**Figure 1:**
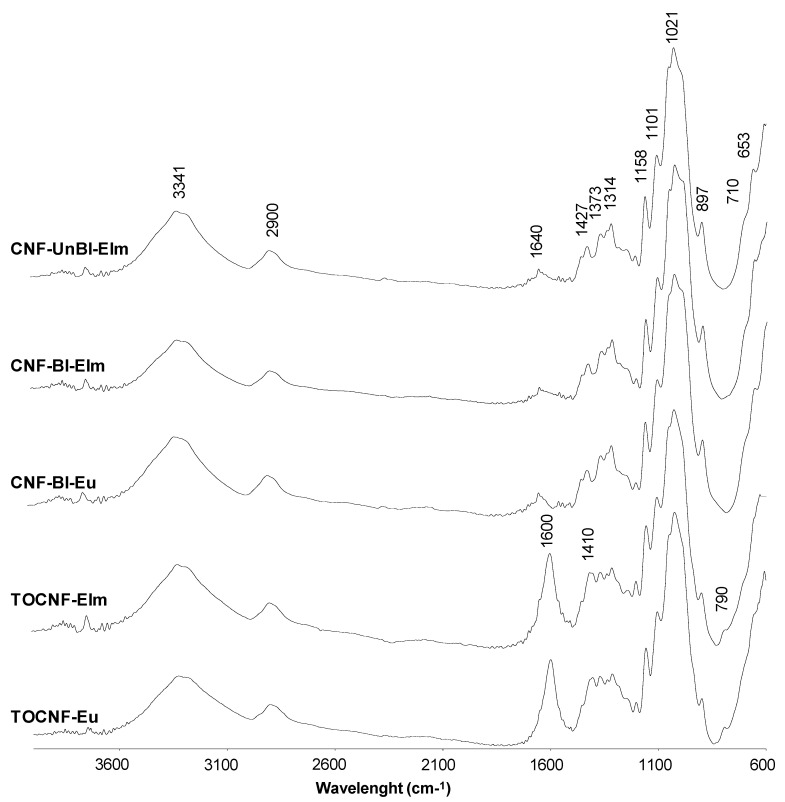
Fourier Transform Infrared (FTIR) spectra of the cellulose nanofiber samples.

FTIR spectra of the five cellulose nanofiber samples (Figure 1) showed the characteristic absorption bands of cellulose [27,28,29,30], including the wide band around 3341 cm^−1^ due to the O–H stretching vibration, and the bands at 2900 and 1427 cm^−1^ corresponding to the C–H stretching vibration and the bending of the –CH_2_ groups, respectively. The band at 1640 cm^−1^ was attributed to the O–H stretching vibration of absorbed water. Bands at 1158, 1101, and 1021 cm^−1^ can be also assigned to cellulose. All vibrations related to pyranosyl rings among C–O asymmetric bands were observed at 1158 cm^−1^, the band at 1101 cm^−1^ corresponded to the C–OH skeletal vibration, while the band at 1021 cm^−1^ was attributed to C–O stretching vibration. The glycosidic bond vibration was detected at 897 cm^−1^. Finally, the peaks at 3280 and 710 cm^−1^ indicated the presence of cellulose Iβ polymorph [30]. 

The main differences between the FTIR spectra of the different cellulose nanofiber samples were related to the pretreatment conditions. Thus, only cellulose nanofibers from chemically pretreated samples (TOCNFs) showed a band at 1600 cm^−1^ associated with the C=O stretching of sodium carboxyl groups [31], due to the oxidation of C6 primary hydroxyl groups during TEMPO-oxidation pretreatment. If these COONa groups were converted into free carboxylic groups (COOH), this peak would shift to 1736 cm^−1^. The band at 1410 cm^−1^ also indicated the presence of COONa, since it is associated to the C–O symmetric stretching of dissociated carboxyl groups [31], although this band has been also assigned to a slight transformation of cellulose I to cellulose II (from 1427 to 1410 cm^−1^) because of the alkaline conditions employed in TEMPO-ox pretreatment [28,32]. Finally, no differences were found between FTIR spectra of CNF-UnBl-Elm and CNF-Bl-Elm because the amount of lignin in unbleached samples was low and, therefore, low intensity lignin peaks would be hidden by the pronounced cellulose bands.

### 3.4. Crystallinity of Cellulose Nanofibers

**Figure 2:**
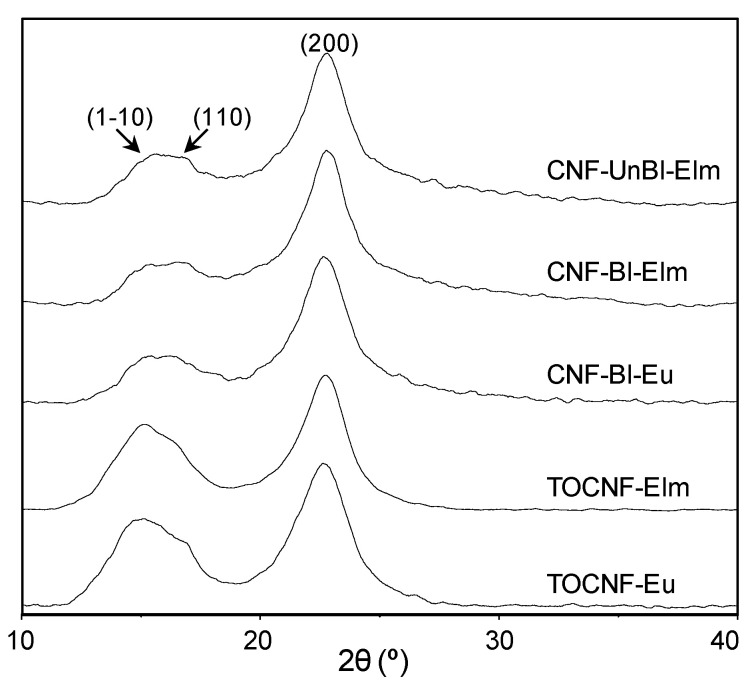
X-ray diffraction patterns of cellulose nanofibers.

X-ray diffractograms of cellulose nanofibers (Figure 2) were used to determine their crystallinity index and crystallite size according to Segal and Scherrer methods, respectively (Table 2). Both crystallinity index and crystallite size depend on the biomass sources [8,9], which justify the higher crystallinity indexes and crystallite sizes observed for elm nanofibers compared to eucalyptus ones. Furthermore, these parameters also depend on the type of pulping, bleaching and nanofibrillation processes which determine not only the degree of attack of cellulose (peeling and chain scissions) but also the removal of other amorphous biomass components such as hemicellulose, lignin and extractives [9,33]. Thus, the higher presence of non-cellulose components in unbleached pulp possibly contributed to the lower crystallinity index of CNF-UnBl-Elm compared to CNF-Bl-Elm (84% vs. 86%, respectively), and the lower crystallite size (8.2 vs. 9.8 nm).

In addition, the nanofibrillation process caused a reduction in crystallinity indexes when compared to original bleached pulps: CrI. of 90% and 89% for Bl-Elm and Bl-Eu pulps, respectively, compared to 86–80% for CNF-Bl samples and 84–77% for TOCNF samples. These results are in agreement with a reduction in crystallinity index due to the breakdown of highly ordered crystalline structure and/or hornification of cellulose nanofibers under a high shear rate during nanofibrillation [12,34]. Furthermore, PFI refining during mechanical pretreatment could also contribute to the reduction in crystallinity index [35]. 

Regarding TEMPO-oxidized pretreatment, a higher reduction in both crystallinity index and crystal size was observed. Although several authors have reported no changes in crystal structure of cellulose I after TEMPO-oxidation [6,16], others have observed a reduction in crystallinity index which could be due to oxidation of internal cellulose crystallites [13,32,36]. Furthermore, this higher reduction in TOCNF samples could be also due to an enhancement of the impact of mechanical nanofibrillation on crystallinity when a chemical pretreatment was carried out [27].

### 3.5. Thermal Stability of Cellulose Nanofibers

**Figure 3:**
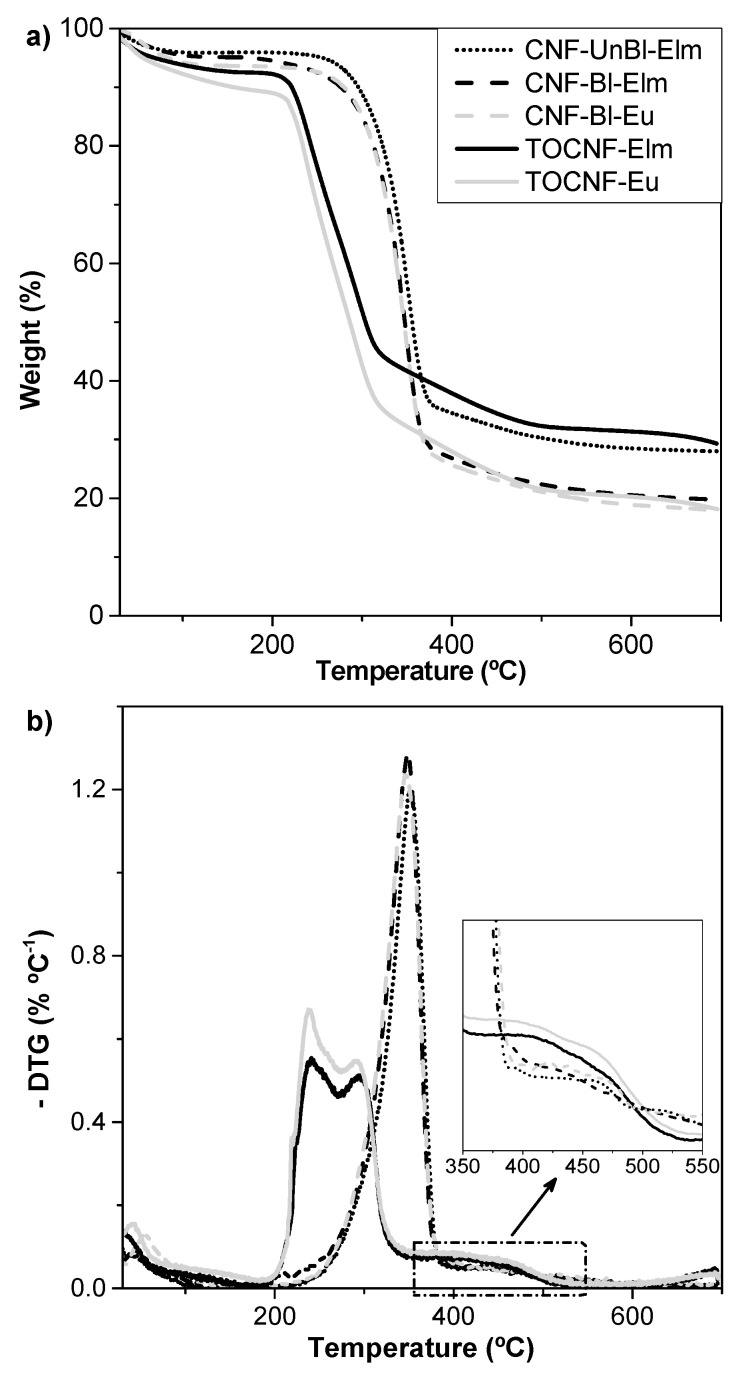
Thermal stability of nanocellulose samples: (**a**) thermogravimetric curves and (**b**) first derivate curves.

A thermogravimetric analysis (TGA) of the cellulose nanofibers was performed to study the thermal stability of the different samples. Thermogravimetric (TG) curves under nitrogen atmosphere and their respective first derivate (DTG) curves are shown in Figure 3, while the main TGA parameters are indicated in Table 3. The major differences observed were due to chemical pretreatment performed on TOCNF samples, which reduced their thermal stability and increased the number of steps in the degradation mechanisms due to decarboxylation of surface carboxyl groups [6,7,27]. Thus, the thermal degradation of CNF samples started at 310–324 °C (T_on_) while TOCNF started to degrade at almost 100 °C earlier (T_on_ of 220–222 °C). DTG curves showed only one peak indicating a degradation temperature (T_deg_) of 347–353 °C for CNF samples, whereas two peaks at 240–241 °C and 291–295 °C and a shoulder at 396–399 °C were appreciated for TOCNF samples. These results are in agreement with those previously reported by Lichtenstein and Lavoine [7], who studied the thermal degradation mechanism of CNF and TOCNF samples with either sodium carboxylate or carboxylic acid surface groups, and confirmed the presence of COONa on the surface of TOCNF (instead of COOH), as also indicated by the FTIR analysis. According to these authors, the thermal degradation mechanism of mechanically pretreated CNF is similar to that of cellulose pulp, occurring in three main stages: (1) 50–150 °C: evaporation of physically adsorbed water, (2) 250–350 °C: breakdown of cellulose glucosidic structure, followed by formation of carbonyl compounds and beginning of aromatization, and (3) 350–600 °C: further char aromatization. However, for chemically pretreated TOCNF samples, four main steps could be distinguished: (1) 50–150 °C: evaporation of physical adsorbed water, (2) 150–250 °C: decarboxylation of sodium carboxylate groups and dehydration of cellulose chains, followed by formation of sodium carbonate and beginning of aromatization process, (3) 250–350 °C: breakdown of cellulose glucosidic structure, further aromatization and formation of sodium carbonate, and (4) 350–600 °C: further aromatization and formation of sodium carbonate [7]. 

Regarding the influence of lignin content on the thermal stability of nanofibrillated cellulose, slightly higher T_deg_, T_on_ and T_off_ were found for CNF-UnBl-Elm compared to CNF-Bl-Elm. This slightly higher thermal stability could be attributed to the presence of aromatic groups, ether and carbon–carbon bonds from residual lignin, which usually decompose at a higher temperature range (250–600 °C) [37]. Nevertheless, the amount of residual lignin on unbleached samples was only 2.5%, explaining that only a slight increase in thermal stability was observed.

Finally, it is worth mentioning the high amount of char residue obtained for all samples. Other authors have also obtained higher char residue than expected considering that cellulose is almost completely degraded after pyrolysis, and hemicellulose (the second main component on the studied pulps) leaves a maximum of 20% of residue [38]. However, according to Claro et al. [38], since hemicellulose degraded at lower temperatures than cellulose, their presence on the surfaces of celluloses nanofibrils could form a coal residue on the cellulose fibrils causing a shielding effect and hindering the complete cellulose degradation. This would explain the higher char residue observed for CNF-UnBl-Elm compared to CNF-Bl-Elm, since unbleached samples presented higher hemicellulose content. On the other hand, TOCNF samples, containing COONa groups, have been reported to leave high char residues, due to the formation of sodium carbonate from the chemical reaction between carbon dioxide (produced during decarboxylation) and water (producing during dehydration) [7]. This fact would explain the higher char residue when comparing TOCNF-Elm to CNF-Bl-Elm (32% vs. 26%, respectively) and TOCNF-Eu to CNF-Bl-Eu (21% vs. 18%, respectively). 

### 3.6. Nanopapers Properties

#### 3.6.1. Mechanical Properties

Nanopapers were formed from nanofibril samples, and their mechanical strength was determined by a tensile test. Independently of raw material, lignin content and pretreatment, all nanopapers showed tensile properties within the typical ranges for cellulose nanofibril films: tensile strength of 56–97 MPa (typically 30–250 MPa), elastic modulus of 4.9–8.5 GPa (typically 1–18 GPa) and elongation to break of 1.1–2.5% (typically 1–10%) [11,39]. 

**Figure 4:**
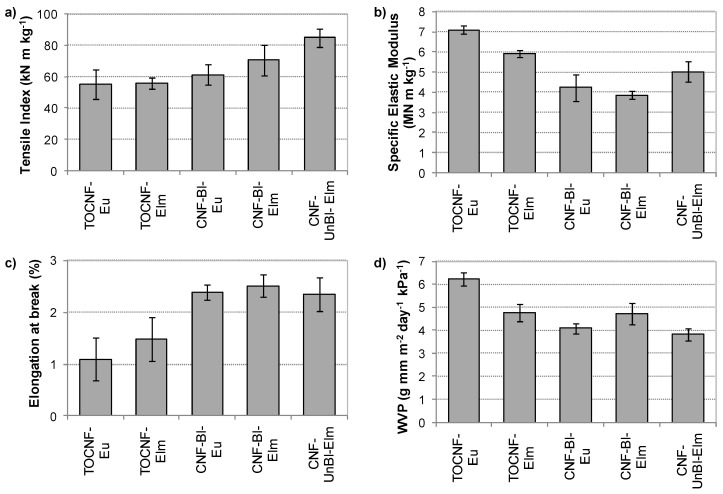
Tensile index (**a**), specific elastic modulus (**b**), elongation to break (**c**) and water vapor permeability (**d**) of nanopapers.

To allow a better comparison between different nanopapers, both tensile index and specific elastic modulus were calculated taking into account the apparent density of the nanopapers. Results are shown in Figure 4, along with elongation to break and water vapor permeability (discussed in the following section). TEMPO-oxidized samples presented lower tensile indexes (55–56 kN m kg^−1^ vs. 61–85 kN m kg^−1^) and elongation to break (1.1–1.5% vs. 2.3–2.5%) than mechanically pretreated samples. These results could be due to the lower degree of polymerization (according to Shinoda et al. [40]) and crystallinity index of TOCNF [41]. Furthermore, the presence of carboxylate groups on the surface of TOCNF, which cannot form hydrogen bonds with either hydroxyl groups or carboxylate groups, reducing the interfibril bonding [6] could also contribute to lower mechanical strength. On the contrary, TOCNF presented much higher nanofibrillation yields (meaning higher amount of nanofibrils), and therefore a higher surface area available for bonding, which would improve the specific elastic modulus [39]. 

When nanopapers from unbleached and bleached samples were compared, higher mechanical properties were found for lignin-containing nanofibers: tensile index of 85 vs. 71 kN m kg^−1^, and specific elastic modulus of 5.0 vs. 3.9 MN m kg^−1^, for CNF-UnBl-Elm and CNF-Bl-Elm, respectively. Similar enhancement of mechanical properties has been previously published for unbleached nanofibrils by Ferrer et al. [18] and Rojo et al. [11]. These authors reported that lignin can improve fibrillation, as previously mentioned, resulting in finer nanofibrils with higher specific surface areas which enhances structure compaction and bonding in the final nanopapers. In our work, although no higher nanofibrillation yield was observed for CNF-UnBl-Elm, this sample showed smaller nanofibrils, as mentioned above. Furthermore, unbleached pulp presented higher hemicellulose content, which can enable strong fiber–fiber bonding upon drying [18], contributing also to enhance mechanical strength. Regarding elongation to break, a slightly lower value was found for unbleached pulp, which could be related to a loss of ductility associated to lignin content, according to Rojo et al. [11].

Finally, the effect of the selected raw material on mechanical properties was not clear. When mechanically pretreated nanofibrils were compared, a similar specific elastic modulus (3.9 vs. 4.2 MN m kg^−1^) and slightly higher tensile index (71 vs. 61 kN m kg^−1^) were found for CNF-Bl-Elm compared to CNF-Bl-Eu, probably due to the higher bonding area caused by a higher nanofibril yield and smaller nanofibrils for the elm sample. On the other hand, for chemically pretreated samples, a lower specific elastic modulus was found for TOCNF-Elm compared to TOCNF-Eu (5.9 vs. 7.1 MN m kg^−1^), while tensile index remained similar (56 vs. 55 kN m kg^−1^). In this case, although TOCNF-Elm presented smaller nanofibrils, which could increase bonding area, it also had higher carboxylate groups content, which could reduce the effective hydrogen bonds between nanofibrils, as mentioned above.

#### 3.6.2. Water Vapor Sorption Isotherms

**Figure 5:**
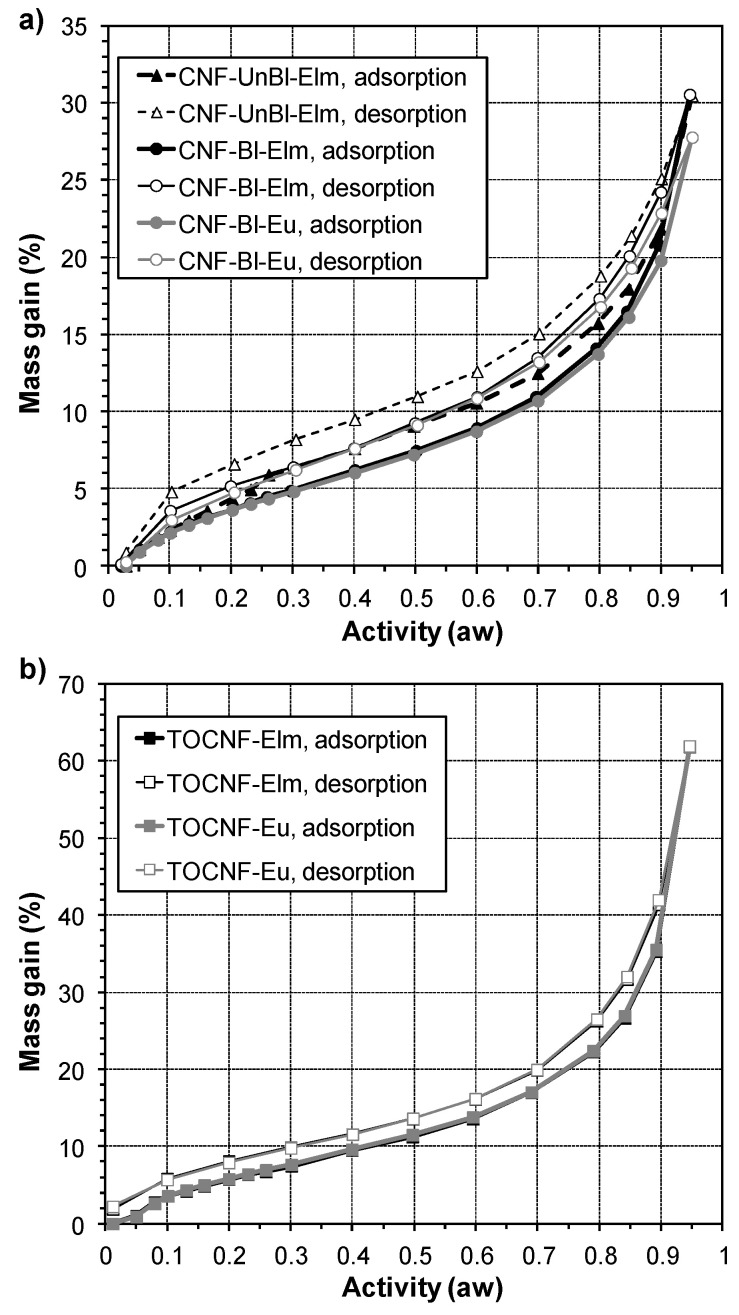
Water sorption and desorption isotherms of the different nanocellulose samples at 25 °C.

The interaction of cellulose nanofibers with water vapor is one of the most important factors affecting the mechanical and barrier properties of nanofiber films. For this reason, the water vapor sorption behavior of the different cellulose nanopapers was studied. Figure 5 shows the sorption and desorption isotherms for mechanically pretreated and chemically pretreated cellulose nanofibrils as a function of water activity (a_w_ = RH/100). For all samples analyzed both sorption and desorption curves displayed a sigmoid or S-shape profile corresponding to type II in the IUPAC classification. This shape is typically observed for cellulose-based materials and in general for hydrophilic materials [42]. The main difference observed was a higher water uptake or equilibrium moisture content (EMC) for TOCNF (62%) compared to CNF (28–31%) samples. This fact is due not only to the higher surface area of TOCNF samples (higher nanofibrillation yield), but also to their stronger water affinity caused by the presence of carboxylate groups on the surfaces of these fibrils, in agreement with previous reports [43,44,45]. These chemically pretreated samples also showed higher sorption hysteresis, especially at high RH (Supplementary Materials, Figure S2). These data indicated a higher swelling of TOCNF samples, compared to CNF, likely due to the lower crystallinity index of TEMPO-oxidized samples, in agreement with the results reported by Guo et al. [45].

On the other hand, a slight difference between CNF-UnBl-Elm and CNF-Bl-Elm isotherm curves was observed, indicating a higher sorption of water in unbleached samples between 20% and 90% RH. This result could be due to lower crystallinity, higher hemicellulose content and/or higher surface area (although unbleached sample showed lower nanofibrillation yield, this nanofibrillated fraction showed lower diameters, as mentioned above). However, at the highest RH (95%), a similar EMC was observed for both samples. 

To better evaluate the water vapor sorption behavior, sorption isotherms were fitted to GAB and Park models. These models are two of the most used for cellulosic materials [42]. More information about the models as well as the correlation curves, fitting data (regression coefficient R^2^) and calculated parameters are shown in the supplementary materials (Figures S3 and S4 and Table S1). In general, both models indicate a higher affinity of water for sorption sites and higher amount of water in the monolayer in TOCNF samples, due to the presence of carboxylate groups [44,45] and the higher surface area. Furthermore, both models indicated a weaker interaction between the surface and water in the monolayer for the CNF-UnBl-Elm sample, probably due to the presence of residual lignin. Finally, it is worth mentioning the higher affinity and strength of bound water to binding sites in elm samples, compared to eucalyptus ones, indicated for both models in TOCNF and CNF-Bl samples.

#### 3.6.3. Barrier Properties

High barrier properties, specially for oxygen gas, have been reported for films prepared from different cellulose nanofibers, due to their capacity to form dense layers and their high crystallinity [8,10]. However, when relative humidity increases, a considerable decrease in barrier properties is observed, due to the adsorption of water and film swelling [8]. For this reason, it is fundamental to study the interaction of nanopapers with water vapor (as mentioned above) and their water vapor permeability. Thus, the water vapor transmission rate (WVTR) was determined for the different nanopapers and the corresponding water vapor permeability values are shown in Figure 4d. Independently of the nanofiber samples used, all nanopapers presented a WVTR in the range 825–1334 g m^−2^ day^−1^ at 75% RH. Comparison of these values with those previously reported is complicated, since not only the relative humidity during the test should be taken into account, but also the method of film preparation. Thus, film casting tends to form more porous films while pressure filtration followed by hot-pressing produces denser films with higher barrier properties [19,46]. Therefore, WVTR of the studied cellulose nanofibers, could be improved if nanopapers were prepared by filtration and hot-pressing.

Figure 4d showed higher WVP for TOCNF nanopapers compared to CNF (4.8–6.2 g mm m^−2^ day^−1^ kPa^−1^ vs. 3.8–4.7 g mm m^−2^ day^−1^ kPa^−1^), especially for TOCNF-Eu, which means lower barrier properties. Chemical pretreatment not only reduced the crystallinity index, which curtails barrier properties [8], but also introduced carboxylate groups on the surface of the nanofibrils, which increase the affinity to water of the fibrils, as observed from water vapor isotherms (higher associated Park’s and GAB’s parameters: bL and KH and CG, Table S1, Supplementary Materials). Furthermore, carboxylate groups also reduce the interfibril bonds between fibrils, contributing to the lower packaging and easier swelling of nanopapers. In concordance, a higher swelling of TOCNF fibrils was indicated by higher hysteresis in Figure 4 and higher Park’s model parameters for water aggregation (Ka and n values in Table S1). Finally, the higher WVP observed for TOCNF-Eu, compared to TOCNF-Elm could be due to a lower crystallinity index and also to the smaller size of TOCNF-Elm nanofibrils, which contributes to more dense nanopapers, improving barrier properties of elm nanopapers.

When mechanically pretreated nanopapers were compared, lower WVP was observed for unbleached sample: 3.8 vs. 5.3 g mm m^−2^ day^−1^ kPa^−1^ for CNF-UnBl-Elm and CNF-Bl-Elm, respectively. In agreement, higher barrier properties have been found for lignin-containing nanofibrils by Rojo et al. [11]. These authors attributed these results to the higher hydrophobicity of unbleached nanopapers (due to lignin presence) and to a reduction in the size and number of micropores due to the reduction in the diameter of the fibrils and the cementing effect of lignin (owing to its softening during the hot-pressing at 100 °C). Thus, although we did not perform hot-pressing of the nanopapers, and CNF-UnBl-Elm showed a lower nanofibrillating yield, this sample presented nanofibrils with lower sizes which could fill the voids between the microfibrils, improving the compaction of the nanopaper. Finally, comparing CNF-Bl-Elm and CNF-Bl-Eu, better barrier properties were observed for eucalyptus samples, contrary to that observed for TOCNF samples.

#### 3.6.4. Optical Properties

**Figure 6:**
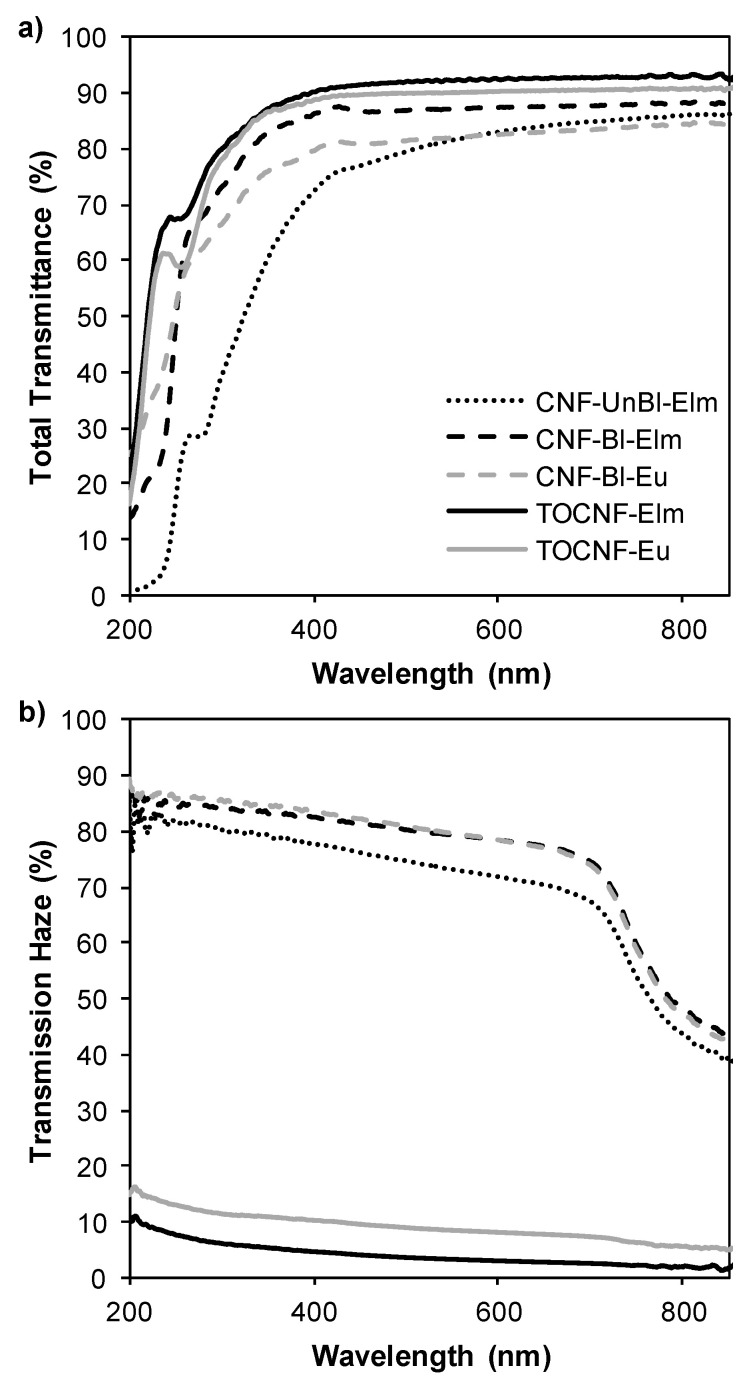
Optical properties of nanopapers: (**a**) total transmittance and (**b**) transmission haze.

Contrarily to conventional paper, nanopapers made only from cellulose nanofibers can be optically transparent if the nanofibers are densely packed and the interstices between the fibers are small enough to avoid light scattering [47]. According to transmittance measurements (Figure 6a), all nanopapers prepared from elm and eucalyptus nanofibrils showed high transparency: transmittance at 600 nm was higher than 83%. As expected, TOCNF nanopapers presented the highest values of transmittance (>90% at 600 nm) due to their high nanofibrillation yield (100%). Nevertheless, although mechanically pretreated samples showed a nanofibrillation yield of 45–61%, these nanopapers also showed high transmittance, observing a correlation with the nanofibrillation yield among CNF nanopapers (CNF-Bl-Eu < CNF-UnBl-Elm < CNF-Bl-Elm). In spite of the presence of microsized fragments, the pores in the bulk paper are still significantly suppressed due to the extensive collapse of the fiber cell wall after microfluidization and the infiltration of nanofibrils in the void spaces of the paper [48]. It is worth mentioning the reduction in transmittance observed for CNF-UnBl-Elm nanopapers at wavelengths lower than 400nm. Thus, lignin-containing nanopapers showed relative UV-protection properties, due to the ability of lignin to absorb UV light owing to its phenolic structure. According to the literature, increasing the lignin content will increase the UV-protection properties [49].

Interestingly, transparent nanopapers have demonstrated better light management than plastic substrates by showing both high transparency and tunable transmission haze [47]. Transmission haze can be defined as the percent of the transmitted beam of light that deviates from the incident beam by more than 2.5° due to light scattering within the bulk of the film [48]. Different strategies have been employed to tune the haze of transparent nanopapers, such as mixing variable amounts of cellulose nanofibrils with microsized TEMPO-oxidized fibers (higher amount of nanofibrils results in lower haze) [48], changing the dispersion solvent (increasing the ratio of ethanol:water increases the transmission haze due to inhomogeneous dispersion of cellulose nanofibrils) [47] or performing mechanical fibrillation with different nanofibrillation yields (weak nanofibrillation increases haze). In agreement, TOCNF samples showed low transmission haze (3–8% at 600 nm), while CNF with lower nanofibrillation yields (45–61%) showed high transmission haze (72–79% at 600 nm). The presence of microsized-fibers (non-fibrillated or partially fibrillated fibers) in CNF nanopapers increased the transmission haze because these microsized fragments show a hollow cylindrical shape that becomes flat in the nanopapers causing light scattering by the refractive index difference between air and cellulose in the compressed cavities, accordingly to Hsieh et al. [50].

The tunable haze of transparent nanopapers increases their applications in optoelectronic devices. Thus, mechanically pretreated nanopapers (CNFs) with high haze would render diffuse scattered light, beneficial in devices such as solar cells, and uniform light distribution for LED lighting or backlight units in a Liquid Crystal Display (LCD). Moderate transmission haze would provide anti-glaring, ensuring comfortable visibility for outdoor devices such as touch panels. On the other hand, TOCNF nanopapers, with low transmission haze, would provide high clarity which is desirable in displays in order to achieve vivid and clear images [48].

## 4. Conclusions

The potential of a DED-resistant *U. minor* cultivar as raw material for the production of cellulose nanofibers has been proved for the first time. Comparing to eucalyptus, easier fibrillation was observed, obtaining nanofibers with lower size and consequently higher barrier and optical properties. Higher tensile indexes were also found for mechanically pretreated elm CNFs. Compared to TEMPO-oxidation pretreatment, mechanical pretreatment provided CNFs with a lower nanofibrillation yield but higher thermal, mechanical and barrier properties, especially for lignin-containing elm pulps. These results support the possibility of using this raw material as feedstock in a lignocellulosic biorefinery.

## Figures and Tables

**Table 1:** Chemical composition of initial pulps, expressed in weight percentage (%).

	Ethanol Extractives	Klason Lignin	Acid Soluble Lignin	Total Lignin	Glucan	Xylan
**UnBl-Elm**	1.3 ± 0.1	1.6 ± 0.1	0.9 ± 0.0	2.5 ± 0.1	75.4 ± 2.3	18.4 ± 0.5
**Bl-Elm**	0.9 ± 0.1	0.9 ± 0.0	0.5 ± 0.1	1.4 ± 0.1	74.7 ± 1.8	17.0 ± 0.9
**Bl-Eu**	0.4 ± 0.2	0.6 ± 0.3	0.7 ± 0.1	1.3 ± 0.3	74.2 ± 0.9	19.3 ± 0.4

**Table 2:** Characteristics of nanofibrillated samples: carboxylate groups content (Carbox.), nanofibrillation yield (Y_Nano_), transmittance at 600 nm (T_600_, suspension at 0.1%), zeta-potential (Z-Pot.), crystallinity index (CrI) and crystallite size (CrS).

	Carbox. (µmol/g)	Y_Nano_ (%)	T_600_ (%)	Z-Pot. (mV)	CrI (%)	CrS (nm)
**CNF-UnBl-Elm**	56 ± 4	50 ± 2	14 ± 2	−7 ± 1	84 ± 1	8.2 ± 0.1
**CNF-Bl-Elm**	53 ± 9	61 ± 3	19 ± 1	−28 ± 1	86 ± 3	9.8 ± 0.2
**CNF-Bl-Eu**	60 ± 7	45 ± 3	15 ± 2	−10 ± 1	80 ± 6	9.2 ± 0.6
**TOCNF-Elm**	1178 ± 37	100 ± 0	93 ± 2	−68 ± 1	84 ± 2	9.4 ± 0.2
**TOCNF-Eu**	1043 ± 150	100 ± 1	77 ± 3	−68 ± 2	77 ± 2	8.2 ± 0.5

**Table 3:** Thermogravimetric analysis (TGA) characteristic parameters for nanocellulose samples: degradation temperature (T_deg_), temperatures at which degradation begins (T_on_) and ends (T_off_), and char residue (ChR).

	T_deg_ (°C)	T_on_ (°C)	T_off_ (°C)	ChR (%)
**CNF-UnBl-Elm**	353	316	373	33
**CNF-Bl-Elm**	350	310	370	26
**CNF-Bl-Eu**	347	312	369	18
**TOCNF-Elm**	241/295/396	220	476	32
**TOCNF-Eu**	240/291/399	222	484	21

## Supplementary Materials

The supplementary materials are available online at https://www.mdpi.com/2073-4360/12/11/2450/s1, Figure S1: Cellulose nanofiber morphology, including dimensions of nanofibers in nanofibrillated fractions of TOCNF and CNF samples, Figure S2: Water vapor sorption hysteresis, Figure S3: Correlation curves and fitting data of water vapor isotherms for GAB model, Figure S4: Correlation curves and fitting data of water vapor isotherms for Park model, Table S1: Sorption parameters of Park and GAB models, determined from water vapor sorption isotherms.

## References

[1] Martín J.A., Sobrino-Plata J., Rodríguez-Calcerrada J., Collada C., Gil L. (2019). Breeding and scientific advances in the fight against Dutch elm disease: Will they allow the use of elms in forest restoration?. New For..

[2] Santini A., Pecori F., Pepori A.L., Ferrini F., Ghelardini L. (2010). Genotype × environment interaction and growth stability of several elm clones resistant to Dutch elm disease. For. Ecol. Manag..

[3] Martin J.A., Solla A., Venturas M., Collada C., Dominguez J., Miranda E., Fuentes P., Buron M., Iglesias S., Gil L. (2015). Seven Ulmus minor clones tolerant to Ophiostoma novo-ulmi registered as forest reproductive material in Spain. iForest Biogeosci. For..

[4] Martínez-Arias C., Sobrino-Plata J., Macaya-Sanz D., Aguirre N.M., Collada C., Gil L., Martín J.A., Rodríguez-Calcerrada J. (2020). Changes in plant function and root mycobiome caused by flood and drought in a riparian tree. Tree Physiol..

[5] Martín-Sampedro R., Eugenio M.E., Fillat Ú., Martín J.A., Aranda P., Ruiz-Hitzky E., Ibarra D., Wicklein B. (2019). Biorefinery of Lignocellulosic Biomass from an Elm Clone: Production of Fermentable Sugars and Lignin-Derived Biochar for Energy and Environmental Applications. Energy Technol..

[6] Isogai A., Saito T., Fukuzumi H. (2011). TEMPO-oxidized cellulose nanofibers. Nanoscale.

[7] Lichtenstein K., Lavoine N. (2017). Toward a deeper understanding of the thermal degradation mechanism of nanocellulose. Polym. Degrad. Stab..

[8] Lavoine N., Desloges I., Dufresne A., Bras J. (2012). Microfibrillated cellulose—Its barrier properties and applications in cellulosic materials: A review. Carbohydr. Polym..

[9] Jonoobi M., Oladi R., Davoudpour Y., Oksman K., Dufresne A., Hamzeh Y., Davoodi R. (2015). Different preparation methods and properties of nanostructured cellulose from various natural resources and residues: A review. Cellulose.

[10] Nechyporchuk O., Belgacem M.N., Bras J. (2016). Production of cellulose nanofibrils: A review of recent advances. Ind. Crops Prod..

[11] Rojo E., Peresin M.S., Sampson W.W., Hoeger I.C., Vartiainen J., Laine J., Rojas O.J. (2015). Comprehensive elucidation of the effect of residual lignin on the physical, barrier, mechanical and surface properties of nanocellulose films. Green Chem..

[12] Liu C., Li B., Du H., Lv D., Zhang Y., Yu G., Mu X., Peng H. (2016). Properties of nanocellulose isolated from corncob residue using sulfuric acid, formic acid, oxidative and mechanical methods. Carbohydr. Polym..

[13] Jiang F., Hsieh Y.-L. (2013). Chemically and mechanically isolated nanocellulose and their self-assembled structures. Carbohydr. Polym..

[14] Solala I., Volperts A., Andersone A., Dizhbite T., Mironova-Ulmane N., Vehniäinen A., Pere J., Vuorinen T. (2011). Mechanoradical formation and its effects on birch kraft pulp during the preparation of nanofibrillated cellulose with Masuko refining. Holzforschung.

[15] NREL (2011). TP-510-42618—Determination of Structural Carbohydrates and Lignin in Biomass.

[16] Saito T., Isogai A. (2004). TEMPO-Mediated Oxidation of Native Cellulose. The Effect of Oxidation Conditions on Chemical and Crystal Structures of the Water-Insoluble Fractions. Biomacromolecules.

[17] Besbes I., Alila S., Boufi S. (2011). Nanofibrillated cellulose from TEMPO-oxidized eucalyptus fibres: Effect of the carboxyl content. Carbohydr. Polym..

[18] Ferrer A., Quintana E., Filpponen I., Solala I., Vidal V., Rodríguez A., Laine J., Rojas O.J. (2012). Effect of Residual Lignin and Heteropolysaccharides in Nanofibrillar Cellulose and Nanopaper. Cellulose.

[19] Solala I., Iglesias M.C., Peresin M.S. (2020). On the potential of lignin-containing cellulose nanofibrils (LCNFs): A review on properties and applications. Cellulose.

[20] Martin-Sampedro R., Revilla E., Villar J.C., Eugenio M.E. (2014). Enhancement of enzymatic saccharification of Eucalyptus globulus: Steam explosion versus steam treatment. Bioresour. Technol..

[21] Wollboldt R.P., Zuckerstätter G., Weber H.K., Larsson P.T., Sixta H. (2010). Accessibility, reactivity and supramolecular structure of *E*. globulus pulps with reduced xylan content. Wood Sci. Technol..

[22] Okita Y., Saito T., Isogai A. (2009). TEMPO-mediated oxidation of softwood thermomechanical pulp. Holzforschung.

[23] Gharehkhani S., Sadeghinezhad E., Kazi S.N., Yarmand H., Badarudin A., Safaei M.R., Zubir M.N.M. (2015). Basic effects of pulp refining on fiber properties—A review. Carbohydr. Polym..

[24] Jacobs A., Larsson P.T., Dahlman O. (2001). Distribution of Uronic Acids in Xylans from Various Species of Soft- and Hardwood As Determined by MALDI Mass Spectrometry. Biomacromolecules.

[25] Okita Y., Saito T., Isogai A. (2010). Entire Surface Oxidation of Various Cellulose Microfibrils by TEMPO-Mediated Oxidation. Biomacromolecules.

[26] Esteban L.G., Guindeo A. (1990). Anatomía de las Maderas de Frondosas Españolas.

[27] Eyholzer C., Bordeanu N., Lopez-Suevos F., Rentsch D., Zimmermann T., Oksman K. (2010). Preparation and characterization of water-redispersible nanofibrillated cellulose in powder form. Cellulose.

[28] Carrillo F., Colom X., Suñol J.J., Saurina J. (2004). Structural FTIR analysis and thermal characterisation of lyocell and viscose-type fibres. Eur. Polym. J..

[29] Oh S.Y., Yoo D.I., Shin Y., Seo G. (2005). FTIR analysis of cellulose treated with sodium hydroxide and carbon dioxide. Carbohydr. Res..

[30] Sugiyama J., Persson J., Chanzy H. (1991). Combined infrared and electron diffraction study of the polymorphism of native celluloses. Macromolecules.

[31] Fujisawa S., Okita Y., Fukuzumi H., Saito T., Isogai A. (2011). Preparation and characterization of TEMPO-oxidized cellulose nanofibril films with free carboxyl groups. Carbohydr. Polym..

[32] Fillat Ú., Wicklein B., Martín-Sampedro R., Ibarra D., Ruiz-Hitzky E., Valencia C., Sarrión A., Castro E., Eugenio M.E. (2018). Assessing cellulose nanofiber production from olive tree pruning residue. Carbohydr. Polym..

[33] Agarwal U.P., Reiner R.R., Ralph S.A. Cellulose crystallinity of woods, wood pulps, and agricultural fibers by FT-Raman spectroscopy. Proceedings of the 16th International Symposium on Wood, Fiber and Pulping Chemistry—Proceedings ISWFPC.

[34] Iwamoto S., Nakagaito A.N., Yano H. (2007). Nano-fibrillation of pulp fibers for the processing of transparent nanocomposites. Appl. Phys. A.

[35] Xu H., Li B., Mu X., Yu G., Liu C., Zhang Y., Wang H. (2014). Quantitative characterization of the impact of pulp refining on enzymatic saccharification of the alkaline pretreated corn stover. Bioresour. Technol..

[36] Meng F., Wang G., Du X., Wang Z., Xu S., Zhang Y. (2019). Extraction and characterization of cellulose nanofibers and nanocrystals from liquefied banana pseudo-stem residue. Compos. Part B.

[37] Imani M., Ghasemian A., Dehghani-Firouzabadi M.R., Afra E., Borghei M., Johansson L.S., Gane P.A.C., Rojas O.J. (2019). Coupling Nanofibril Lateral Size and Residual Lignin to Tailor the Properties of Lignocellulose Films. Adv. Mater. Interfaces.

[38] Claro F.C., Matos M., Jordão C., Avelino F., Lomonaco D., Magalhães W.L.E. (2019). Enhanced microfibrillated cellulose-based film by controlling the hemicellulose content and MFC rheology. Carbohydr. Polym..

[39] Xu J., Krietemeyer E.F., Boddu V.M., Liu S.X., Liu W.-C. (2018). Production and characterization of cellulose nanofibril (CNF) from agricultural waste corn stover. Carbohydr. Polym..

[40] Shinoda R., Saito T., Okita Y., Isogai A. (2012). Relationship between Length and Degree of Polymerization of TEMPO-Oxidized Cellulose Nanofibrils. Biomacromolecules.

[41] Fukuzumi H., Saito T., Isogai A. (2013). Influence of TEMPO-oxidized cellulose nanofibril length on film properties. Carbohydr. Polym..

[42] Belbekhouche S., Bras J., Siqueira G., Chappey C., Lebrun L., Khelifi B., Marais S., Dufresne A. (2011). Water sorption behavior and gas barrier properties of cellulose whiskers and microfibrils films. Carbohydr. Polym..

[43] Meriçer Ç., Minelli M., Giacinti Baschetti M., Lindström T. (2017). Water sorption in microfibrillated cellulose (MFC): The effect of temperature and pretreatment. Carbohydr. Polym..

[44] Lundahl M.J., Cunha A.G., Rojo E., Papageorgiou A.C., Rautkari L., Arboleda J.C., Rojas O.J. (2016). Strength and Water Interactions of Cellulose I Filaments Wet-Spun from Cellulose Nanofibril Hydrogels. Sci. Rep..

[45] Guo X., Liu L., Hu Y., Wu Y. (2018). Water vapor sorption properties of TEMPO oxidized and sulfuric acid treated cellulose nanocrystal films. Carbohydr. Polym..

[46] Österberg M., Vartiainen J., Lucenius J., Hippi U., Seppälä J., Serimaa R., Laine J. (2013). A Fast Method to Produce Strong NFC Films as a Platform for Barrier and Functional Materials. ACS Appl. Mater. Interfaces.

[47] Jiang F., Li T., Li Y., Zhang Y., Gong A., Dai J., Hitz E., Luo W., Hu L. (2018). Wood-Based Nanotechnologies toward Sustainability. Adv. Mater..

[48] Fang Z., Zhu H., Bao W., Preston C., Liu Z., Dai J., Li Y., Hu L. (2014). Highly transparent paper with tunable haze for green electronics. Energy Environ. Sci..

[49] Sadeghifar H., Venditti R., Jur J., Gorga R.E., Pawlak J.J. (2017). Cellulose-Lignin Biodegradable and Flexible UV Protection Film. ACS Sustain. Chem. Eng..

[50] Hsieh M.-C., Koga H., Suganuma K., Nogi M. (2017). Hazy Transparent Cellulose Nanopaper. Sci. Rep..

