# Supplementary material for: Cellulose Nanofibers from a Dutch Elm Disease-Resistant Ulmus minor Clone
Source: Polymers (Basel). 2020 Oct 23;12(11):2450. doi: 10.3390/polym12112450 (PMC7690703; doi:10.3390/polym12112450)
Supplement: Supplementary file 1 [file polymers-12-02450-s001.pdf]

Supplementary Materials for:

Cellulose nanofibers from a dutch elm disease resistant *Ulmus minor* clone

Laura Jiménez-López[1,2], María E. Eugenio[1], David Ibarra[1], Margarita Darder[2], Juan A. Martín[3], Raquel Martín-Sampedro[1,2,*]

[1] Forestry Products Department, Forest Research Centre, INIA, Ctra de la Coruña Km 7.5, Madrid 28040, Spain. mariaeugenia@inia.es (M.E.E.), ibarra.david@inia.es (D.I), raquel.martin@inia.es (R.M-S.)

[2] Instituto de Ciencia de Materiales de Madrid (ICMM), Consejo Superior de Investigaciones Científicas (CSIC), Sor Juana Inés de la Cruz 3, Cantoblanco, Madrid 28049, Spain. laura.jimenez.lopez@csic.es (L.J-L.), darder@icmm.csic.es (M.D.)

[3] Departamento de Sistemas y Recursos Naturales, ETSI Montes, Forestal y del Medio Natural, Universidad Politécnica de Madrid, c. José Antonio Novais 10, Madrid 28040, Spain. juan.martin.garcia@upm.es (J.A.M.)

* Correspondence: raquel.martin@inia.es.

1. CELLULOSE NANOFIBERS MORPHOLOGY

Morphology of individual nanofiber was studied by atomic force microscopy (AFM) Cervantes instrument (Nanotec Electrónica S.L., Spain) under ambient conditions using Nanosensors PPP-FMR cantilevers with a spring constant of 2.8 N/m and a resonance frequency of 75 KHz. Images were obtained in Amplitude Modulation Mode with oscillation amplitude of 10 nm. Before measurement, nanofibers were diluted and centrifuged at 6000 rpm during 5 minutes, to remove non-fibrillated and partially-fibrillated fibers. A drop of the supernatant was deposited on a mica platelet, previously modified by (3-aminopropy)triethoxysilane [1]. After 30 s of incubation the mica platelet was thoroughly rinsed with deionized water and blown dry with N_2. Images were processed with WSxM 4.0 Beta 9.3 from WSxM solutions, to determine diameter and length of the nanofibers.

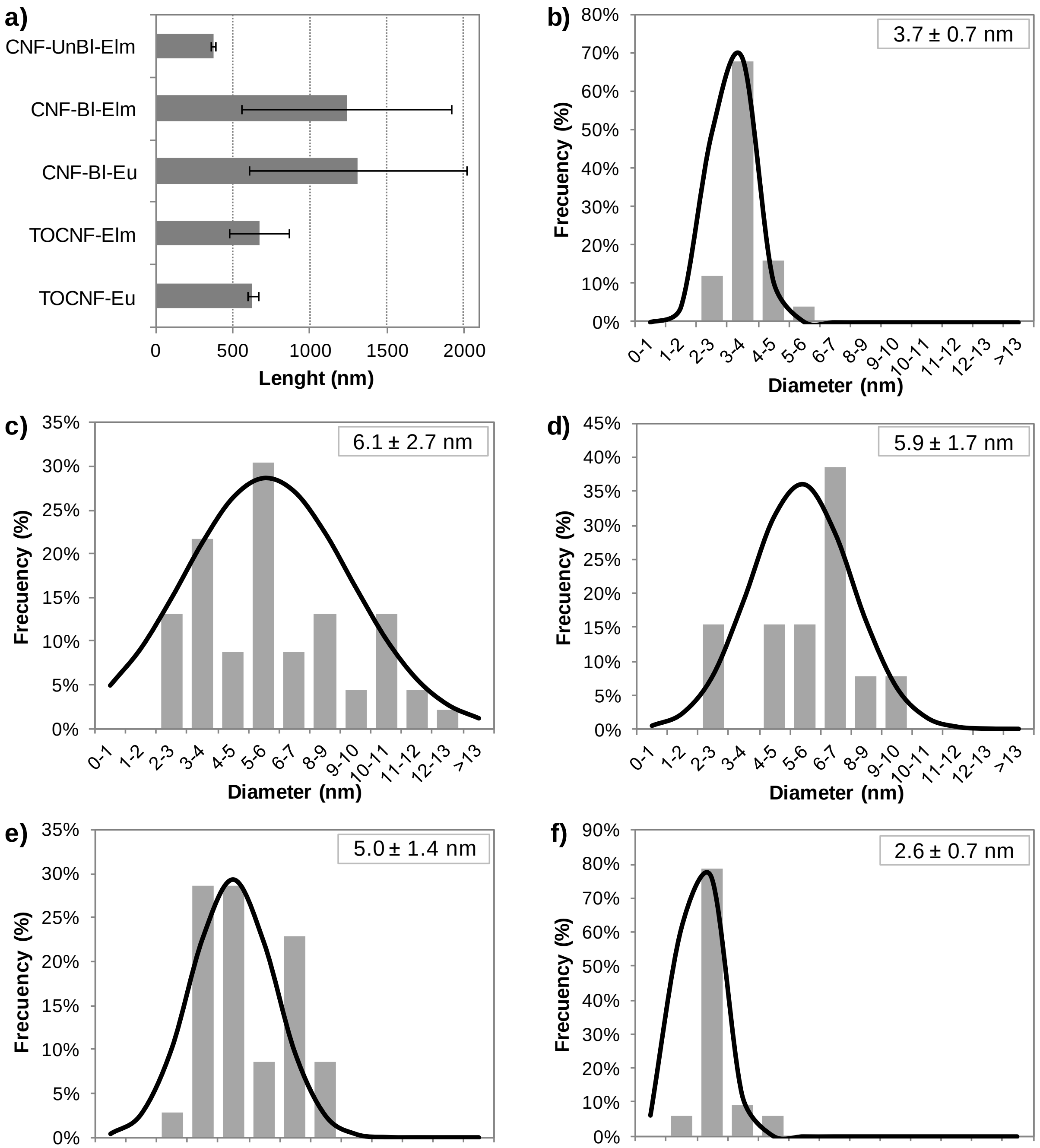

Figure S1. Dimensions of nanofibers in nanofibrillated fraction of TOCNF and CNF samples: lengths (a) and diameters of CNF-UnBl-Elm (b), CNF-Bl-Eu (c), CNF-Bl-Elm (d), TOCNF-Eu (e), and TOCNF-Elm (f).

Figure S1 shows the length and diameter distribution of the nanofibers in the nanofibrillated fraction of TOCNF and CNF samples (after removing non-fibrillated and partially fibrillated fibrils by centrifugation). Chemical pretreatment originated shorter and thinner nanofibers than mechanical pretreatment, as expected. It can be also observed the presence of thinner nanofibers in elm samples compared to eucalyptus ones: lower diameter and narrow diameter distribution in both TOCNF and CNF elm samples. Furthermore thinner and shorter nanofibers were observed for CNF-UnBl-Elm (diameter of 3.7 ± 0.7 nm and length of 370 ± 14 nm) compared to CNF-Bl-Elm (5.9 ± 1.7 nm and 1237 ± 680 nm). Short nanofibers were also found in TOCNF samples, as expected due to chemical pretreatment.

2. WATER VAPOR SORPTION HYSTERESIS

Figure S2 shows sorption hysteresis of the different cellulose nanopaper samples. A higher sorption hysteresis was found for samples chemically pretreated, especially at high relative humidity.

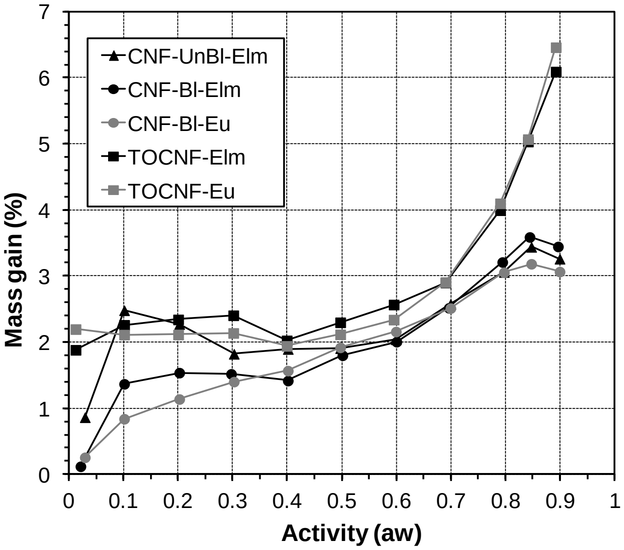

Figure S2. Sorption hysteresis of the different nanopapers.

3. WATER VAPOR SORPTION MODELS

To better evaluate the water vapor sorption behavior, sorption isotherms were fitted to GAB and Park models. These models are two of the most used for cellulosic materials [2].

GAB model considers that water molecules adsorb layer by layer on adsorption surface (external surfaces of specific sites or internal surfaces of micro-pores/cavities). The first layer of water covers the surface unevenly and is tightly bound in a monolayer. Subsequent layers display increasingly bulk-like properties. The total water uptake (C_{GAB}) is calculated as function of water activity (a_w) according to equation S1, where: C_m is defined as the monolayer capacity and represents the amount of water adsorbed onto one layer; K_{ads} refers to the adsorption enthalpy difference between the first layer and the following; and C_G is the Guggenheim constant which measures the strength of bound water to the primary binding sites.

$$C_{GAB} = C_m \cdot \frac{C_G \cdot K_{ads} \cdot a_w}{(1 - K_{ads} \cdot a_w) \cdot (1 + (C_G - 1) \cdot K_{ads} \cdot a_w)} \qquad \text{(Eq S1)}$$

Park model assumes the association of three sorption mechanisms: 1) Langmuir sorption, describing the specific sorption of water molecules on specific sites (ions and polar groups, such as hydroxyl groups, from external surface, amorphous regions, inner surface of voids and crystallites) [2]; 2) non-specific sorption which increase linearly with water activity (Henry's law); and 3) water molecules aggregation (clustering) at high water activity. Thus, the water uptake (C_{Park}) is calculated by the sum of the three terms, according to equation S2, where: A_L is the Langmuir capacity constant, related to the concentration of the specific sorption sites; b_L is the Langmuir affinity constant of water for these sorption sites; K_H is the Henry's solubility coefficient, K_a is the equilibrium constant for the clustering reaction; and n is the average number of water molecules per cluster.

$$C_{Park} = \frac{A_L \cdot b_L \cdot a_w}{1 + b_L \cdot a_w} + K_H \cdot a_w + K_a \cdot n \cdot a_w^n \qquad \text{(Eq S2)}$$

Figure S3 and S4 shows correlation curves and fitting data (regression coefficient R^2) for each nanopaper sample for GAG and Park models, respectively. Regression coefficient (R^2) indicated that both models are consistent with the experimental data in the whole water activity range, but Park model showed better accuracy.

Calculated parameters for both Park and GAB models are shown in Table S1. Comparing TOCNF and CNF samples, Park model described similar concentration of specific sorption sites (A_L) in both types of samples, in spite of the higher surface area expected for TOCNF samples (higher nanofibrillation yield). Nevertheless, this model did indicate higher affinity of water for sorption sites in TOCNF samples (higher b_L), due to the presence of carboxylate groups [3,4]. In

agreement, GAB model indicated higher strength of bound water with primary binding sites (C_G) for TOCNF samples, and also higher amount of water in the monolayer (C_m), likely due to higher surface area. Between $0.1 < a_w < 0.6$, the higher Henry's coefficient (K_H) found for TOCNF samples, indicated higher adsorption in amorphous, interfibrillar regions [5], in concordance with the lower crystallinity index and higher swelling found for TOCNF samples. These last characteristics of TOCNF samples are likely the reason of the higher clustering equilibrium (Ka) and the higher size of clusters (n) found for chemically pretreated samples.

According to Park model, CNF-UnBl-Elm sample presented an unexpected higher amount of specific binding sites (A_L), compared to CNF-Bl-Elm, which could be due to the small size of nanofibrils in this sample or to the contribution of hemicelluloses, among other factors. The higher surface area could be also the reason of the higher amount of water adsorbed in the monolayer according to GAB model (C_m). Both models indicated a weaker interaction between the surface and water in the monolayer (lower b_L and C_G) for CNF-UnBl-Elm sample, probably due to the presence of residual lignin. Differences in the rest of the parameters were less significant between bleached and unbleached samples. Finally, it is worth mentioning the higher affinity (b_L) and strength of bound water to binding sites (C_G) in elm samples, compared to eucalyptus ones, indicated for both models in TOCNF and CNF-Bl samples.

Table S1: Sorption parameters of Park and GAB models, determined from water vapor sorption isotherms.

	Park model					GAB model		
	A_L	b_L	K_H	K_a	n	C_m	C_G	K_{ads}
**CNF-UnBl-Elm**	7.5	1.8	11.5	2.2	13	5.2	9.9	0.9
**CNF-Bl-Elm**	1.2	12.4	13.6	2.6	13	4.0	11.9	0.9
**CNF-Bl-Eu**	1.7	6.9	12.4	2.3	11	4.2	9.9	0.9
**TOCNF-Elm**	1.1	21.3	22.5	6.0	16	5.4	17.2	1.0
**TOCNF-Eu**	1.3	16.5	22.5	6.0	16	5.5	16.8	1.0

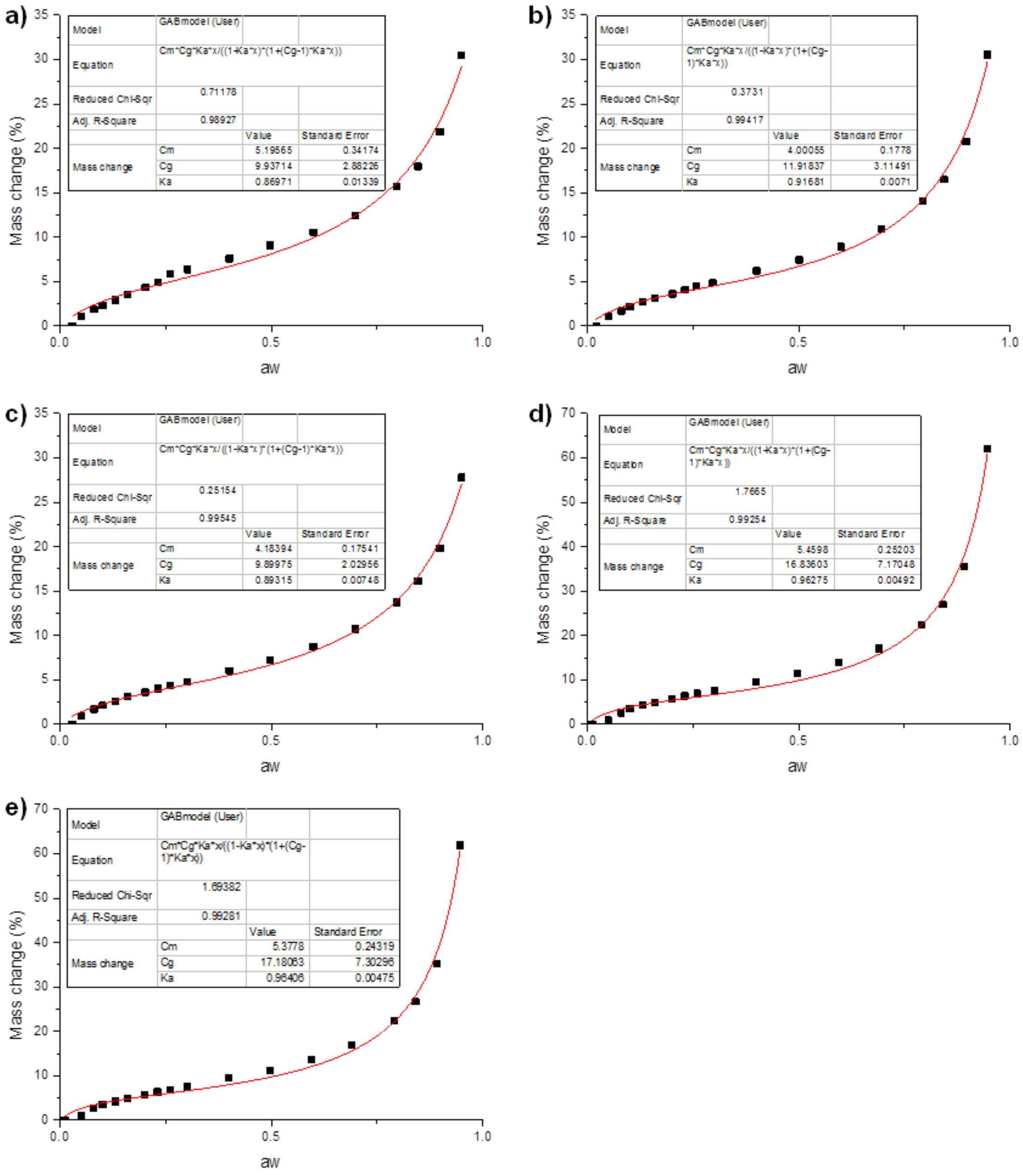

Figure S3. Correlation curves and fitting data of water vapor isotherms for GAB model: CNF-UnBl-Elm (a), CNF-Bl-Eu (b), CNF-Bl-Elm (c), TOCNF-Eu (d), and TOCNF-Elm (e).

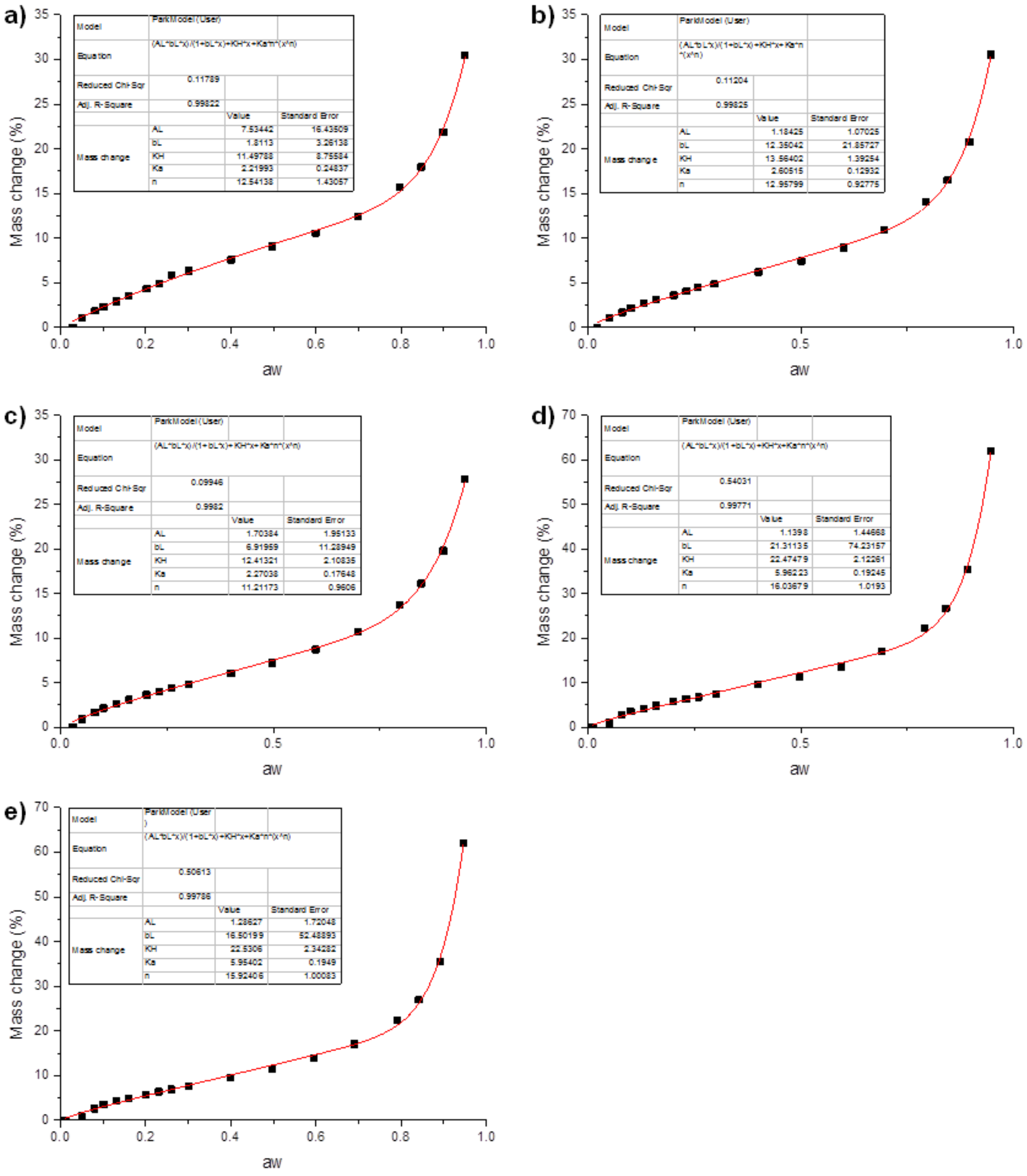

Figure S4. Correlation curves and fitting data of water vapor isotherms for Park model: CNF-UnBl-Elm (a), CNF-Bl-Eu (b), CNF-Bl-Elm (c), TOCNF-Eu (d), and TOCNF-Elm (e).

REFERENCES

1. Usov, I.; Nyström, G.; Adamcik, J.; Handschin, S.; Schütz, C.; Fall, A.; Bergström, L.; Mezzenga, R. Understanding nanocellulose chirality and structure–properties relationship at the single fibril level. *Nat. Commun.* **2015**, *6*, 7564, doi:10.1038/ncomms8564.

2. Belbekhouche, S.; Bras, J.; Siqueira, G.; Chappey, C.; Lebrun, L.; Khelifi, B.; Marais, S.; Dufresne, A. Water sorption behavior and gas barrier properties of cellulose whiskers and microfibrils films. *Carbohydr. Polym.* **2011**, *83*, 1740–1748, doi:https://doi.org/10.1016/j.carbpol.2010.10.036.

3. Lundahl, M.J.; Cunha, A.G.; Rojo, E.; Papageorgiou, A.C.; Rautkari, L.; Arboleda, J.C.; Rojas, O.J. Strength and Water Interactions of Cellulose I Filaments Wet-Spun from Cellulose Nanofibril Hydrogels. *Sci. Rep.* **2016**, *6*, 30695, doi:10.1038/srep30695.

4. Guo, X.; Liu, L.; Hu, Y.; Wu, Y. Water vapor sorption properties of TEMPO oxidized and sulfuric acid treated cellulose nanocrystal films. *Carbohydr. Polym.* **2018**, *197*, 524–530, doi:https://doi.org/10.1016/j.carbpol.2018.06.027.

5. Meriçer, Ç.; Minelli, M.; Giacinti Baschetti, M.; Lindström, T. Water sorption in microfibrillated cellulose (MFC): The effect of temperature and pretreatment. *Carbohydr. Polym.* **2017**, *174*, 1201–1212, doi:https://doi.org/10.1016/j.carbpol.2017.07.023.
